# *Bursaphelenchus xylophilus* detection and analysis system based on CRISPR – Cas12

**DOI:** 10.3389/fpls.2022.1075838

**Published:** 2022-12-15

**Authors:** Xiang Wang, Lai-Fa Wang, Ye-Fan Cao, Yan-Zhi Yuan, Jian Hu, Zu-Hai Chen, Fei Zhu, Xi-Zhuo Wang

**Affiliations:** ^1^ Key Laboratory of Forest Protection of National Forestry and Grassland Administration, Ecology and Nature Conservation Institute, Chinese Academy of Forestry, Beijing, China; ^2^ Jingning County Forest Resources Management Center, Lishui, China; ^3^ Hangzhou Linping District Forest Resources Protection and Management Station, Hangzhou, China

**Keywords:** *Bursaphelenchus xylophilus*, nucleic acid detection, CRISPR-Cas12a, LAMP, rapid diagnostic

## Abstract

Pine wilt disease is caused by the pine wood nematode (*Bursaphelenchus xylophilus*) and leads to wilting and death of pines. It is one of the most damaging diseases of pines worldwide. Therefore, accurate and rapid detection methods are of great importance for the control of *B. xylophilus*. Traditional detection methods have some problems, such as being time-consuming and requiring expensive instruments. In this study, the loop-mediated isothermal amplification (LAMP) and clustered regularly interspaced short palindromic repeats (CRISPR) were used to establish a set of intelligent detection and analysis system for *B. xylophilus*, called LAMP-CRISPR/Cas12a analysis, which integrated field sampling, rapid detection and intelligent control analysis. The process can be completed within 1 hour, from sample pretreatment and detection to data analysis. Compared with the single LAMP method, the LAMP-CRISPR/Cas12a assay uses species-specific fluorescence cleavage to detect target amplicons. This process confirms the amplicon identity, thereby avoiding false-positive results from non-specific amplicons, and the large amounts of irrelevant background DNA do not interfere with the reaction. The LAMP-CRISPR/Cas12a assay was applied to 46 pine wood samples and the samples carrying *B. xylophilus* nematodes were successfully identified. To meet the needs of different environments, we designed three methods to interpret the data: 1) naked eye interpretation; 2) lateral flow biosensor assay; and 3) integrated molecular analysis system to standardize and intellectualize the detection process. Application of the *B. xylophilus* detection and analysis system will reduce the professional and technical requirements for the operating environment and operators and help to ensure the accuracy of the detection results, which is important in grass-root *B. xylophilus* detection institutions.

## Introduction

The pine wood nematode, *Bursaphelenchus xylophilus*, is the causal agent of pine wilt disease. *B. xylophilus* is listed as a quarantine pest in more than 40 countries (Zhao et al., 2009). Many pines die from pine wilt disease, which substantially damages economies and ecosystems. Current methods for the treatment of pine wilt disease are not ideal, so early detection and prevention have become the primary methods for managing the disease.

Detection of *B. xylophilus* has been based mainly on morphological and molecular identification. Morphological identification is time-consuming and requires a high level of taxonomical expertise (Floyd et al., 2002; Kikuchi et al., 2009; Cha et al., 2019), and can sometimes be difficult or impossible because species in genus *Bursaphelenchus* have similar morphologies (Kang et al., 2004).

Molecular identification is more accurate than morphological identification, but standard identification methods such as PCR assays require expensive equipment and take more time. Isothermal amplification-based detection technologies have overcome the limitations associated with PCR-based assays (Zhao et al., 2015). Isothermal amplification approaches, such as loop-mediated isothermal amplification (LAMP) and recombinant polymerase amplification (RPA), have been successfully applied to detect *B. xylophilus*. These approaches have eliminated the need for thermocycling instruments and can be used for real-time detection, thereby supporting field and point-of-care testing (Zhou et al., 2022; Meng et al., 2022). However, these technologies generally are unable to distinguish nucleotide sequences that differ in only one, or even several, bases; they need to be optimized, and may result in false positives because of non-specific amplification, cross-contamination, and/or primer dimerization (Wang et al., 2018; Joung et al., 2020). Therefore, a fast, sensitive, and highly specific diagnostic platform for nucleic acid detection is still needed.

Clustered regularly interspaced short palindromic repeats (CRISPRs) are DNA sequences found in prokaryotic genomes (Barrangou, 2015). The CRISPR-associated immune system is exploited in molecular biology to target and cleave specific nucleic acid sequences and is typically used in gene editing. Additionally, after binding to the target double-stranded DNA (dsDNA) or RNA, several CRISPR proteins can be activated to release non-specific endoribonuclease activity for the degradation of single-stranded DNA (ssDNA) and RNA, thereby providing a novel diagnostic method for nucleic acid detection (Abudayyeh et al., 2017; Chen et al., 2018; Gootenberg et al., 2018; Myhrvold et al., 2018; Chertow, 2018). The Cas12 single RNA-guided endonuclease has been used for the CRISPR diagnosis targets dsDNA and ssDNA. Cas12 requires a protospacer adjacent motif site in the target region for dsDNA cleavage and sieving ssDNA (Yan et al., 2019). Cas12a was first reported in 2018 (Chen et al., 2018). The DETECTR detection method directs Cas12a of bacteria in family Lachnospiraceae or other organisms to dsDNA targets through its complementary CRISPR RNA (crRNA), triggering collateral cleavage of short ssDNA reporters that carry a fluorophore and a quencher. Target recognition and reporter cleavage lead to separation of the quencher from the fluorophore, which produces a fluorescent signal. DETECTR attains attomolar sensitivity when combined with RPA. Besides DETECTR, other Cas12a-based detection technologies have been developed, such as HOLMES (one-hour, low-cost, multipurpose, highly efficient system), which uses PCR and Cas12a of Lachnospiraceae for preamplification (Li et al., 2018a; Li et al., 2018b), and HOLMESv2 (Li et al., 2019), which uses LAMP combined with a thermostable Cas12b from *Alicyclobacillus acidoterrestris* in a one-pot reaction. HOLMES and HOLMESv2 have a detection limit of approximately 10 aM. Cas12f also targets dsDNA and ssDNA and is better than Cas12a at discriminating single-nucleotide polymorphisms in ssDNA (Harrington et al., 2018). The Bio-SCAN toolkit uses RPA and CRISPR to make the Bio-SCAN as an attractive molecular diagnostic tool for diverse populations applications in agriculture (Sánchez et al., 2022). The CRISPR technology is already being used in a variety of different ways. For example, testing for pathogens bacteria (Zhang et al., 2021), authentication of halal food (Wu et al., 2021), and detection of transgenic crops (Zhu et al., 2022). CRISPR technology has a promising application prospect in the detection of pathogenic agents, Mu et al. report a CRISPR-Cas12a-based nucleic acid assay for an early and rapid diagnosis of wheat Fusarium head blight (Mu et al., 2022), Xia et al. developed a label-free assay for *Salmonella enterica* detection based on the G-quadruplex-probing CRISPR-Cas12 system (termed G-CRISPR-Cas), allowing highly sensitive detection of *S. enterica* and investigation of their colonization in chickens (Xia et al., 2021). Aman et al. report the development of a simple, rapid, and efficient RT-RPA method, coupled with a CRISPR/Cas12a-based one-step detection assay, to detect plant RNA viruses (Aman et al., 2020). Zhang et al. report an assay to directly analyze pathogenic genes based on CRISPR-Cas12 (Zhang et al., 2021). but there are few studies on its application to pine wilt disease caused by *B. xylophilus*.

**Figure 1:**
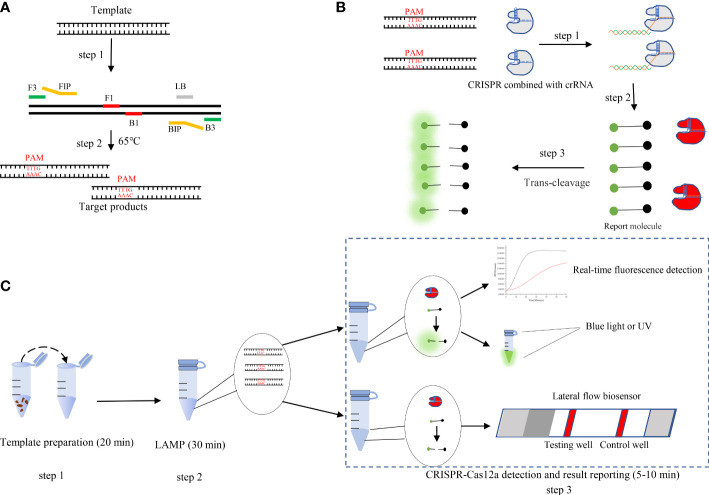
**(A)** Schematic illustration of loop-mediated isothermal amplification (LAMP). **(B)** Schematic illustration of CRISPR-Cas12a detection. Step 1: LAMP products are obtained. Protospacer adjacent motif (PAM) sites guide the CRISPR/Cas12a-gRNA complex to recognize target sites. Step 2: Cas12a effectors are activated. Step 3: The activated effectors nonspecifically cleave single-stranded DNA reporter molecules by trans-cleavage. **(C)** Schematic illustration of the LAMP-CRISPR/Cas12a assay workflow. The LAMP-CRISPR/Cas12a assay involves three closely linked steps: rapid template preparation (step 1), LAMP reaction (step 2), and CRISPR-Cas12a cleavage and signal detection (step 3).

In this study, we developed a novel molecular assay that combines the CRISPR/Cas12a system with LAMP to detect *B. xylophilus* and visualized the combined LAMP-CRISPR/Cas12a assay results. First, we amplified the target DNA using LAMP technology, and then used crRNA to guide CRISPR protein to detect the amplified products. However, when the amplified products was the target, the CRISPR protein would cut the target and be activated to cut the reporter molecule, which produced fluorescence after being cut (**Figure 1**). This molecular assay has the potential to detect *B. xylophilus* with high sensitivity and specificity without the need for expensive experimental equipment, and provides a convenient and straightforward method that can be deployed in the field for rapid *B. xylophilus* detection.

## Materials and methods

### Specimen collection and preparation, and DNA extraction

Wood and nematode samples were collected for DNA extraction.

We sampled pine trees using a wood sampler (Wang et al., 2017), and DNA was extracted from the samples by the Chelex-100 method (Gou et al., 2015). We modified the Chelex-100 by removing the ice bath process and increasing the boiling water bath time, which was improved by taking 50 mg of the log, putting it in a 2 mL centrifuge tube, and adding 400 µL of extraction buffer (3 M·L^−1^ guanidine isothiocyanate [CH5N3·HSCN], 50 mM·L^−1^ Tris-HCl [pH 8.0], 20 mM·L^−1^ EDTA [pH 8.0], and 1% Triton X-100). The mixture was stirred and boiled for 10 min, then 200 µL of 5% (w/v) Chelex-100 was added. The mixture was stirred and incubated in a boiling water bath for 8 min. The purity, quality, and concentration of the extracted DNA were determined using a NanoDrop ND-1000 spectrophotometer (NanoDrop Technologies, USA). The extracted DNA was stored at −20°C.

To collect pure nematode samples, nematodes were isolated from wood samples using the Baermann funnel technique and observed with a binocular microscope (Nikon, SMZ-2, Stereoscopic Zoom Microscope, Japan). The nematode isolates were maintained on cultures of *Botrytis cinerea* grown on potato dextrose agar at 25°C. The prepared nematode cultures were stored in the laboratory until use. Nematode DNA was extracted using a DNA extraction kit (TIANGEN Biotic Company, China).

### Polymerase chain reaction

We selected the *B. xylophilus* synaptogenesis protein gene *SYG-2* as the target gene. The *SYG-2* nucleotide sequences of *B. xylophilus* and *B. mucronatus* showed notable interspecies differences but intraspecies conservation (Gou, 2014). PCR amplification of *SYG-2* was performed using primers SYG2-part2-F and SYG2-part2-R. Each PCR amplification was performed in a final volume of 25 µL comprising 1 µL template DNA, 12.5 µL PCR Mix (New England BioLabs, USA), 1 µL of each 10 µM primer, and 9.5 µL ddH2O, using a T100 Touch PCR (Bio-Rad, USA). The PCR protocol was 94°C for 3 min, followed by 35 cycles at 94°C for 1 min, 53°C for 30 s, and 72°C for 45 s, with a final extension at 72°C for 10 min, followed by storage at 4°C.

### LAMP reaction

The LAMP assay was established using two pairs of primers and total genomic DNA extracted from *B. xylophilus*. The initial conditions of the LAMP reaction were based on those reported previously (Notomi et al., 2000; Gou, 2014). Each reaction was performed in a final volume of 25 µL comprising 2.5 µL of 10× Isothermal Amplification Buffer (New England BioLabs), 1.6 µM of both 4FIP and 4BIP, 0.8 µM of LoopF, 0.2 µM of both 4F3 and 4B3, 1.4 mM dNTPs mix (TIANGEN Biotic Company), 8 U *Bst* 2.0 WarmStart DNA Polymerase (New England BioLabs), 10 mM MgSO_4_ (New England BioLabs), and 2 µL total genomic DNA. To reduce heat transfer and prevent contamination, 20 μl of mineral oil was added to cover the LAMP reaction mixture.The reaction was conducted in a 0.2 mL clear PCR tube at 65°C for 60 min in a water bath. The *Bst* 2.0 WarmStart DNA Polymerase was inactivated at 80°C for 10 min. The LAMP results were interpreted and visualized using SYBRgreenI and hydroxynaphthol blue (HNB) dyes. The primers were designed based on the *SYG-2* nucleotide sequence and were specific for *B. xylophilus* (Gou, 2014).

### Preparation of crRNA

We designed the crRNA using CRISPR-offinder software (Zhao et al., 2017) and targeted a previously identified conserved gene that encodes *SYG-2* crRNA targeting *SYG-2*. The crRNA was complemented to the target sites with a 5′ TTTV protospacer adjacent motif sequence in the DNA strand opposite the target sequence (**Table 1**). The crRNA was synthesized by Integrated DNA Technologies (USA).

**Table 1:** Sequences of LAMP and PCR primers, crRNA, and report ssDNA.

The name of the primer	Primer sequence (5’-3’)
4B3	AAGCGGTCTAAGCGAAAC
4F3	TGTAAACACCGTATAAAGGAATT
4BIP	CCGATTGTCTAACTTCTGCTGCGCTTGTTCTCTGAGACCATA
4FIP	CATCCTTTGGTCGCTTTCTGAGTTTTAAAAATTTCACCACGTT
4LBA syg2-PART2- F syg2-PART2-R	TTCCGAAAAGCTTGGGTAAAATCCT GGTAATATTATTGGAGGAAGGAATATAA TGAGAACTGCTCGACGATCTTG
crRNA-1	AGAAAGCGACCAAAGGAUGGUUG
crRNA-2	GUCGCUUUCUGAAAAAAAUAUUU
crRNA-3 ssDNA-FB ssDNA-LFB	GGAAAAUACAAAAAUAGGCAGCA /56-FAM/TTATT/3BHQ1 /56-FAM/TTATT/3Biotin

### Generation of the LAMP-CRISPR/Cas12a assay

Each Cas12a reaction mixture contained 250 nM Cas12a, 500 nM crRNA, 300 nM ssDNA-fluorophore-quencher (FQ) reporter (FAM-TTATT-BHQ1), 2.5 µL NEBuffer 2.1, and 2 µL PCR product or 2 µL LAMP product. The total volume was adjusted to 25 µL with nuclease-free water. The tubes were incubated at 37°C for 60 min then inactivated at 98°C for 2 min. The LAMP-CRISPR/Cas12a assay was monitored using a LAMPPY DNA detection system (OZ Optics Ltd, Canada). The PCR products were used to demonstrate the feasibility of the method and the LAMP products were used to confirm that binding to LAMP took place. Cas12a of Lachnospiraceae (Lba Cas12a) and NEBuffer 2.1 were purchased from New England Biolabs.

### Lateral flow biosensor assay

For lateral flow detection, 100 µL HybriDetect Assay Buffer and 10 μl aliquot of products from the CRISPR-Cas12a trans-cleavage mixture (250 nM Cas12a, 500 nM crRNA, 400 nM ssDNA-lateral flow biosensor reporter, 2 µL LAMP product, and 2.5 µL NEBuffer 2.1) were added to a reaction tube. A HybriDetect 1 ​​lateral flow dipstick (Milenia Biotec GmbH, Giessen, Germany) was placed in the solution and the tube was incubated for 5–15 min in an upright position. At the end of the incubation period, the dipstick was removed from the assay solution and the test result was interpreted immediately.

### Reaction time of the LAMP-CRISPR/Cas12a assay

After adding *B. xylophilus* genomic DNA, the LAMP reaction was performed at 65°C for 10, 15, 20, 25, 30, 35, 40, 50, or 60 min. LAMP products generated under the different reaction times were collected and added to the Cas12a reaction system. Fluorescence intensity was measured to determine the optimal reaction time.

### Specificity and sensitivity of the LAMP-CRISPR/Cas12a assay

The analytical specificity of the LAMP-CRISPR/Cas12a assay was tested on at least 5 ng of templates extracted from four *B. xylophilus* strains and three related species, *B. mucronatus*, *B. doui*, and *Botrytis cinerea*.

The analytical sensitivity for detecting a decreasing number of gene copies was evaluated using purified *B. xylophilus* DNA concentrations diluted from 10^−1^ to 10^−8^ to give DNA concentrations of 66.4 ng/μL, 6.64 ng/μL, 664 pg/μL, 66.4 pg/μL, 6.64 pg/μL, 0.664 pg/μL, 66.4 fg/μL, and 6.64 fg/μL.

We perform base alterations or deletions at one or more locations of the target DNA sequence to test that CRISPR-Cas12a enhanced fluorescence analysis can distinguish several base changes (**Supplementary Table S1**).

### Interpretation of the LAMP-CRISPR/Cas12a assay results

The LAMP-CRISPR/Cas12a assay results were interpreted in three different ways: 1) the reaction results were irradiated with blue or ultraviolet light, and the green fluorescence was observed by the naked eye; 2) ​​lateral flow dipsticks were used to visualize the experimental results; and 3) a fluorescence detection instrument was used to detect the fluorescence changes in real-time.

### Detection of *B. xylophilus* in field samples by LAMP-CRISPR/Cas12a assay

To assess the ability of the LAMP-CRISPR/Cas12a assay to detect *B. xylophilus* in field samples, we tested 46 samples and compared the results with the results of the PCR assay (**Supplementary Table S2**). The Chelex-100 method was used to extract DNA from the field samples. PCRs were used to verify the detection results. The DNA used for the PCR amplification was extracted using a DNA extraction kit.

## Results

### Optimization of the LAMP-CRISPR/Cas12a assay

**Figure 2:**
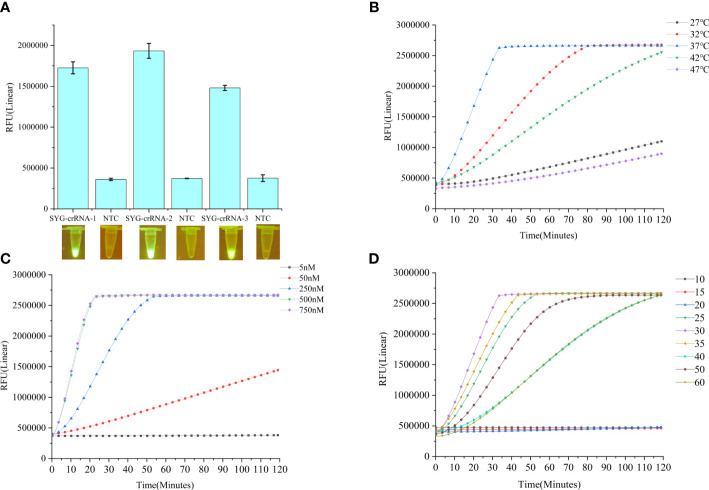
**(A)** Fluorescence changes of three crRNA reactions for 1 h detected by CRISPR-Cas12a fluorescence assays. Values are shown in the graph as means ± SD (n = 3). **(B)** Fluorescence curves at different reaction temperatures. **(C)** Fluorescence curves of different crRNA concentrations. **(D)** Effects of different LAMP reaction times on the Cas12a reaction. NTC: no template control.

In this study, we established a CRISPR–Cas12a enhanced fluorescence assay coupled with PCR amplification to detect *B. xylophilus*. Negative control DNA and *B. xylophilus* genomic DNA were used separately as templates to evaluate the validity of the assay. Blue-light transilluminators were used to determine whether the products could be visualized to detect *B. xylophilus* with the naked eye. A fluorescent signal emitted under blue light reflected cleavage of the FAM-TTATT-BHQ1 reporter. Three crRNAs of *B. xylophilus* were designed and synthesized as *in vitro* transcripts. CRISPR-Cas12a fluorescence assays showed the different detection activities of the three crRNAs. Among these, SYG-crRNA-2 had the highest detection activity for the target PCR product (**Figure 2A**). Therefore, SYG-crRNA-2 was used to enhance the fluorescence intensity for CRISPR-Cas12a-assisted detection of *B. xylophilus* DNA. We also tested the optimal reaction temperature, comparing the reaction rates at 27°C, 32°C, 37°C, 42°C and 47°C, and found that the optimal reaction temperature was 37°C (**Figure 2B**). When the crRNA concentration of the reaction was 5nM, 50nM, 250nM, 500nM and 750nM, it was found that the reaction speed increased with the increase of the concentration, but reached the maximum value at 500nM (**Figure 2C**).

### Optimal reaction time of the LAMP-CRISPR/Cas12a assay

*B. xylophilus* was detected by CRISPR-Cas12a combined with LAMP. The LAMP-CRISPR/Cas12a system combines LAMP and CRISPR-Cas12a detection. The optimal time of the LAMP reaction is crucial for the whole detection system; therefore, we investigated the optimal LAMP reaction time. After addition of *B. xylophilus* DNA, the LAMP reaction was performed for 10, 15, 20, 25, 30, 35, 40, 45, 50, 55, or 60 min, followed by the Cas12a reaction for 120 min. We found that the best LAMP reaction time was 30 min (**Figure 2D**).

### Specificity of the LAMP-CRISPR/Cas12a assay

**Figure 3:**
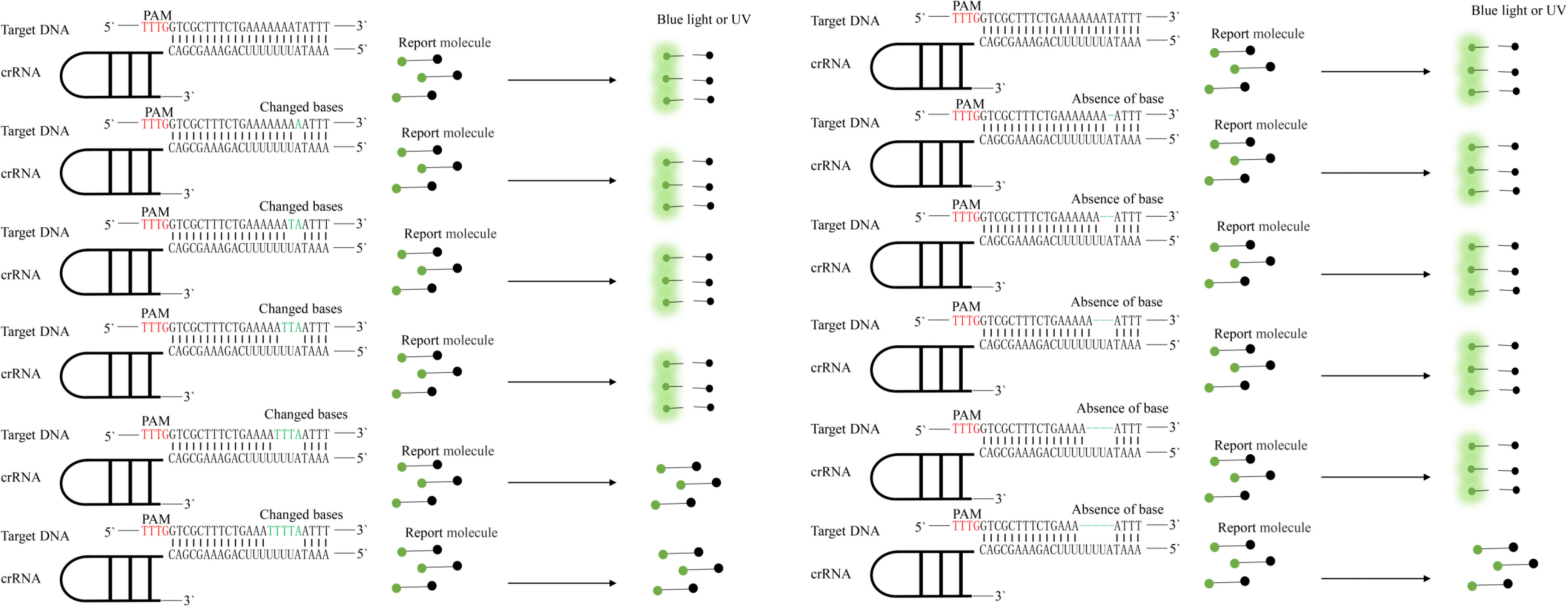
Specificity of the CRISPR-Cas12a enhanced fluorescent assay evaluated by its ability to distinguish base mismatches. Protospacer adjacent motif (PAM) sequences are shown in red; base mismatches are shown in green.

We made base changes or deletions at one or more positions in the target DNA sequence and detected the modified DNA sequences by Cas12a (**Figure 3**).

**Figure 4:**
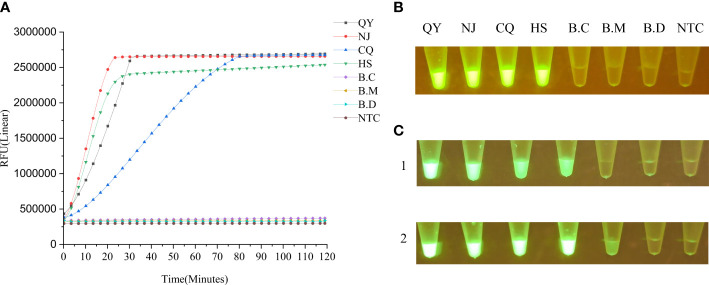
**(A)** Specificity of the LAMP-CRISPR/Cas12a assay evaluated by its ability to distinguish *B xylophilus* strains and related species. B.C: *Botrytis cinerea*; B.M: *B mucronatus*; QY: *B xylophilus* (from Liaoning); NJ: *B xylophilus* (from Nanjing); CQ: *B xylophilus* (from Chongqing); HS: *B xylophilus* (from Anhui); B.D: *B doui*; NTC: negative control (ddH2O). **(B)** End-point fluorescence visualization of the specificity test. **(C)** Detection of base changes or deletions in target sequences by LAMP-CRISPR/Cas12a assay by measuring fluorescence intensity under blue-light irradiation 1 h after the reaction. Panel 1: From left to right, 0–5 base changes; the last tube is the negative control. At four base changes, the fluorescence intensity dropped significantly. Panel 2: From left to right, 0–5 base deletions. the last tube is the negative control. At five base deletions, the fluorescence intensity dropped significantly.

We also explored the specificity of the LAMP-CRISPR/Cas12a assay by comparing the amplification results with two related, *B. mucronatus*, *B. doui*, and one unrelated, *Botrytis cinerea*¸ species. By real-time monitoring of the reaction fluorescence, we found that the LAMP-CRISPR/Cas12a assay detected different *B. xylophilus* strains and accurately distinguished two related, and one unrelated species (**Figures 4A**, **B**). This system also detected base changes or deletions in target sequences and distinguished a four-base difference and a five-base deletion (**Figure 4C**).

### Sensitivity of the LAMP-CRISPR/Cas12a assay

**Figure 5:**
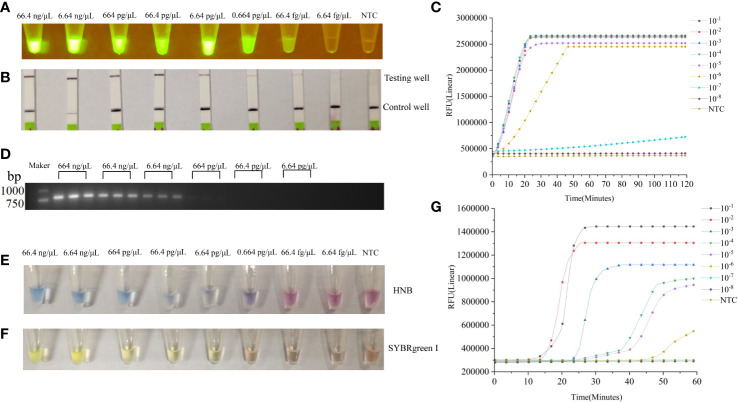
**(A)** Sensitivity of the LAMP-CRISPR/Cas12a assay for *B xylophilus* detection. Purified *B xylophilus* genomic DNA was diluted by 10^−1^, 10^−2^, 10^−3^, 10^−4^, 10^−5^, 10^−6^, 10^−7^, and 10^−8^ to give DNA concentrations of 66.4 ng/µL, 6.64 ng/µL, 664 pg/µL, 66.4 pg/µL, 6.64 pg/µL, 0.664 pg/µL, 66.4 fg/µL, and 6.64 fg/µL. Different concentrations of DNA were detected by LAMP-CRISPR/Cas12a assay and visualized by blue light. **(B)** Results of the LAMP-CRISPR/Cas12a assay visualized by lateral flow biosensor. **(C)** Real-time fluorescence profiles of DNA at different concentrations in LAMP-CRISPR/Cas12a assay. **(D)** Sensitivity of the PCR assay for *B xylophilus* detection. The image shows amplification bands targeting the *SYG-2* gene. Lanes 1–6 lanes are *B xylophilus* genomic DNA dilutions 664 ng/μL, 66.4 ng/µL, 6.64 ng/µL, 664 pg/µL, 66.4 pg/µL, and 6.64 pg/µL (3 replicates per concentration). **(E, F)** Different concentrations of DNA were detected by traditional LAMP and visualized by HNB and SYBRgreenI. **(G)** Real-time fluorescence curves of DNA with different concentrations in LAMP.

Sensitivity of the LAMP-CRISPR/Cas12a assay for *SYG-2* gene detection was compared with that of the PCR assay. The concentration of the extracted DNA was determined to be 664 ng μL^−1^. The analytical sensitivities for detecting decreasing numbers of gene copies were evaluated by diluting the purified DNA of *B. xylophilus* from 10^−1^ to 10^−8^ per reaction. The LAMP-CRISPR/Cas12a assay detection limit was 0.664 pg/µL (**Figure 5C**), but when the reaction time was prolonged, the concentration of 66.4 fg/μL could also produce weak fluorescence (**Figure 5A**). The detection limit of the PCR assay for the same DNA template was 664 pg/µL (**Figure 5D**).

### Comparison between the LAMP-CRISPR/Cas12a assay and the traditional LAMP

We compared LAMP-CRISPR/Cas12a assay with traditional LAMP (**Table 2**). The LAMP-CRISPR/Cas12a assay was used to detect DNA at different concentrations, and the results were visualized by irradiation with blue light (**Figure 5A**). We tested the ability of lateral flow biosensor to visualize the results of the LAMP-CRISPR/Cas12a assay; however, the detection limit of the lateral flow biosensor was not as straightforward as that of direct blue-light irradiation (**Figure 5B**). When the DNA concentration was diluted to 10^-7^, fluorescence was produced, but it took a long time and the fluorescence was not strong. When the DNA concentration was diluted to 10^-8^, no fluorescence was produced (**Figure 5C**). The LAMP results were visualized using HNB dyes (**Figure 5D**) and SYBRgreenI (**Figure 5E**). The results showed that the line between positive and negative became blurred as the concentration decreased, and the use of HNB dyes showed a decrease in color when the concentration was 66.4 pg/μL. When the concentration was 6.64 pg/μL, SYBRgreenI showed a color reduction. Compared with the LAMP-CRISPR/Cas12a assay, although the traditional LAMP also had an amplification curve at 10^-6^ (**Figure 5F**), it was only with the aid of the instrument that amplification could be accurately judged. When using the dye method, the color would not be obvious at 10^-4^, making it difficult to determine whether amplification was possible. Whereas the LAMP-CRISPR/Cas12a assay detected the DNA even at low concentrations, the traditional LAMP failed to distinguish DNA from the negative control at the low DNA concentrations. The CRISPR system cuts up reporter molecules to produce fluorescence that clearly separates positive samples from negative ones. Compared with LAMP, LAMP-CRISPR/Cas12a assay is also more specific for detecting specific amplified sequences, while LAMP only assesses whether a test target is amplified or not, and cannot identify non-specific amplification.

**Table 2:** Comparison of the LAMP-CRISPR/Cas12a Assay with Traditional LAMP for detection of *B. xylophilus*.

Detection methord	Traditional LAMP	LAMP-CRISPR/Cas12a Assay
Assay reaction time	60min	40-50min
Temperature of reaction	65°C	65°C and 37°C
Analysis object	dsDNA, Pyrophosphate ions or Mg2+, etc	dsDNA
Need large instrument or not	No	No
Number of tests	1	2
Assay cost	low	low

### Detection of LAMP-CRISPR/Cas12a assay in field samples

The LAMP-CRISPR/Cas12a assay was used to detect *B. xylophilus* in 46 dead pine samples, and *B. xylophilus* was detected in 38 of them (**Supplementary Table S2**). We verified the results by isolating the nematodes and identifying them using the Baermann funnel method. DNA was extracted from the isolated nematodes and PCR amplifications were performed. The results were consistent with those obtained by the LAMP-CRISPR/Cas12a assay.

## Discussion

**Figure 6:**
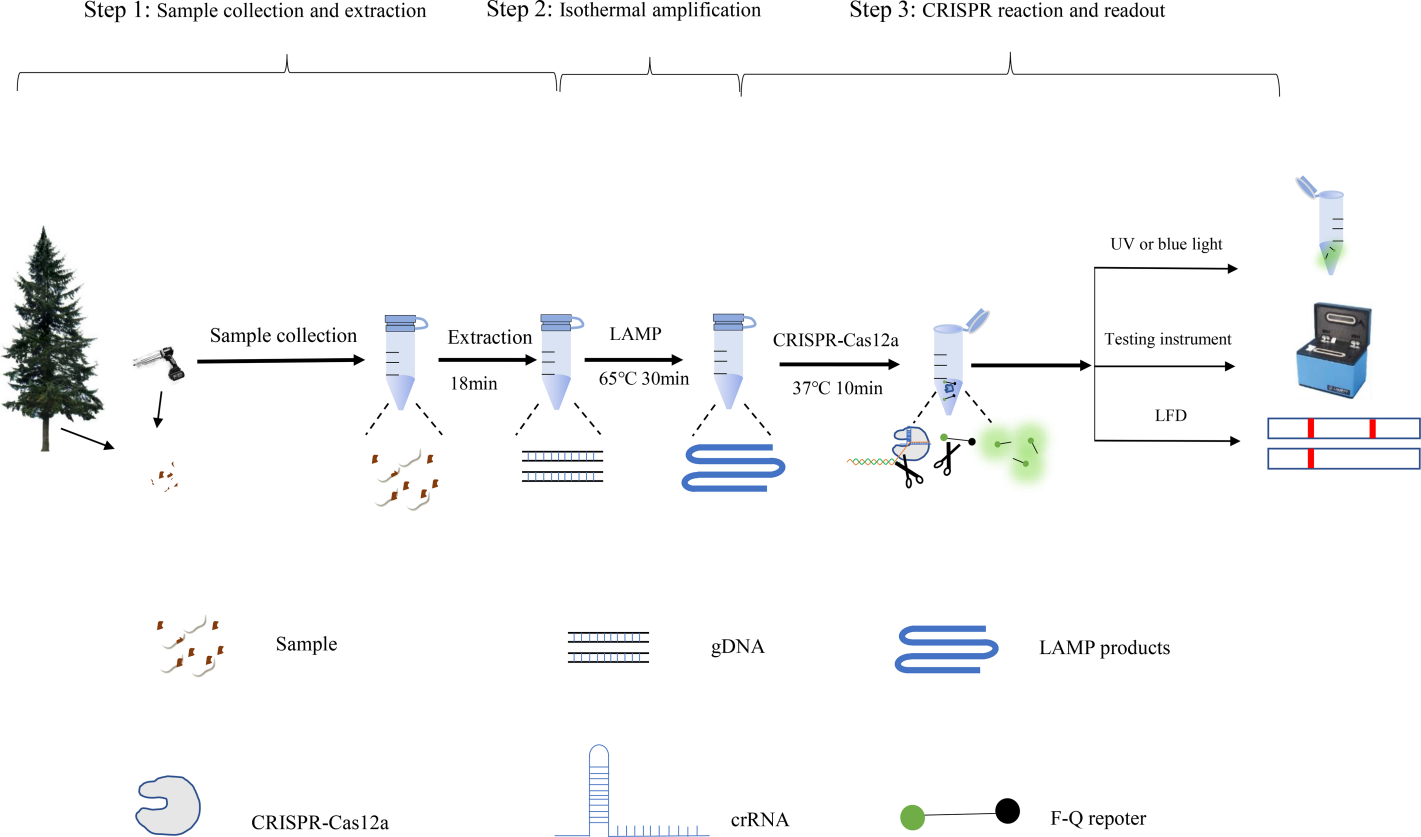
Processing scheme for the LAMP-CRISPR/Cas12a assay includes the following steps: DNA extraction, isothermal amplification reactions, and CRISPR-Cas12 detection of the *B xylophilus* by fluorescence.

The causative agent of pine wilt disease, *B. xylophilus*, is particularly prevalent in China and causes significant economic and ecological losses. Establishing a sensitive, reliable, effective, and rapid diagnostic method is key to detecting *B. xylophilus* and preventing infection. Currently, molecular diagnostic techniques for *B. xylophilus* rely mainly on PCR (Kanzaki and Futai, 2002; Cardoso et al., 2012) and real-time qPCR techniques (Cao et al., 2005; François et al., 2007). Although these techniques have been widely validated and are useful tools for detecting this disease, reducing the detection time and instrument requirements remains a challenge. In this study, we established a convenient, highly sensitive LAMP coupled with CRISPR/Cas12a assay to rapidly detect *B. xylophilus*. The assay requires three steps, DNA extraction, LAMP reaction, and CRISPR detection, which can be completed in 1 h (**Figure 6**).

LAMP (Notomi et al., 2000) is a highly efficient nucleic acid amplification technology that can potentially improve disease diagnosis in plant protection. LAMP technology has been applied for detecting *B. xylophilus* and the methods have constantly been improved (Kikuchi et al., 2009; Leal et al., 2015; Meng et al., 2022). LAMP assays can detect *B. xylophilus* with high sensitivity and efficiency; however, interpretation of LAMP reaction results has been a critical issue. Currently available analysis methods include colorimetric agents, fluorescent agents, agarose gel electrophoresis, and turbidimeters (Zhang et al., 2014), which are not specific to target amplicons and thus cannot differentiate specific and non-specific amplification. We have shown that combining LAMP with the CRISPR system can avoid non-specific amplification.

In this study, we designed three crRNAs that target *SYG-2* for the rapid detection of *B. xylophilus*. The effects of the three crRNAs were not significantly different, with crRNA-2 being the most effective. We compared the specificity of this method for four strains of *B. xylophilus*, and *B. mucronatus*, *B. doui*, and *Botrytis cinerea*. To test whether this method could identify single-base differences in sequences, we changed the target sequence. We found that this method could identify only the change of four bases and the deletion of five bases, which is different from the single base change reported by Gootenberg (Gootenberg et al., 2018). Although single base changes were not identified, the LAMP-CRISPR/Cas12a assay was better than the single LAMP reaction at determining whether the amplified product was a target gene rather than a non-specific amplification.

Considering that LAMP is the first step before the CRISPR reaction and that the LAMP reaction time has a crucial impact on the CRISPR reaction, we tested different LAMP reaction times and found that after LAMP for 30 min, the CRISPR reaction resulted in the fastest growth of the fluorescence value, which reached its maximum at 30 min. The CRISPR reaction process is quite fast, and fluorescence was very obvious at 10 min when the blue-light irradiation showed very obvious green fluorescence.

Interpretation of test results is always a crucial part of the whole testing process. Many methods are available for the interpretation of CRISPR results, including blue and ultraviolet light irradiation and excitation fluorescence. Using a blue-light flashlight or UV flashlight is a simple and convenient method that can provide a straightforward interpretation of the CRISPR results. An instrument for real-time detection can be used to interpret fluorescence curves. The ​​lateral flow dipstick method, which is based on introducing two small molecular markers FAM and biotin at both ends of the probe, has also been used to interpret CRISPR results (Wang et al., 2020; Wei et al., 2022). For this method, the anti-FAM antibody was labeled with colloidal gold, and the detection line on the NC film was coated with streptavidin, which could bind biotin. The concentration of the probe was adjusted to ensure that all the colloidal gold was in the lower line before the probe was cut. Once the probe was cut, the colloidal gold crossed the lower line and reached the upper line for color development. The ​​lateral flow dipstick method does not need an instrument, but the current test strip price is high, which will significantly increase the detection cost.

The cost of detection is a major problem that has restricted the popularization and application of detection methods. For example, the fluorescence quantitative PCR instrument is expensive and recombinant polymerase amplification-​​lateral flow dipsticks are approximately 50 CHY each (Zhou et al., 2022). The DETECTR detection method (Chen et al., 2018) was shown to accurately detect the risk of human papillomavirus (HPV) infection, HPV16 (100%) and HPV18 (92%), and DETECTR tests cost less than one U.S. dollar each. The LAMP-CRISPR/Cas12a detection system that we developed uses simple blue-light irradiation to visualize the results and does not require expensive instruments; a single detection cost less than 15 CHY. Thus, the detection cost was greatly reduced.

However, applying CRISPR detection to *B. xylophilus* diagnosis still has some problems; for example, storage of reagents at room temperature is a significant problem. Lyophilized applications of entire CRISPR reaction systems have been reported (Nguyen et al., 2021; Rybnicky et al., 2022), suggesting that this is where the future application of CRISPR detection should be focused.

## Conclusions

*B. xylophilus* causes pine wilt disease, which is one of the most damaging diseases of pines worldwide. In this study, we established a new method for detecting *B. xylophilus*, which was more sensitive and faster than traditional PCR assays, and effectively avoided false positives caused by unrelated DNA that can appear with the single LAMP method. The developed LAMP-CRISPR/Cas12a assay has a simple read-out system that does not require any special instrumentation and can be performed in areas with minimal laboratory infrastructure, making it an attractive alternative method for *B. xylophilus* detection.

## Data availability statement

The original contributions presented in the study are included in the article/**Supplementary Material**. Further inquiries can be directed to the corresponding author.

## Author contributions

XW and X-ZW conceived the study. XW and YC performed the experiments; JH, ZC, and FZ contributed materials. Y-ZY and WX analyzed the data. X-ZW and L-FW secured funding. XW and X-ZW wrote the manuscript. All authors contributed to the article and approved the submitted version.
