# Supplementary material for: Bursaphelenchus xylophilus detection and analysis system based on CRISPR – Cas12
Source: Front Plant Sci. 2022 Dec 15;13:1075838. doi: 10.3389/fpls.2022.1075838 (PMC9800051; doi:10.3389/fpls.2022.1075838)
Supplement: Supplementary file 1 [file DataSheet_1.docx]

**TABLE S1 Mismatched position in the targeting sequence.**

|  | **Target sequence** |
| --- | --- |
| 1 | 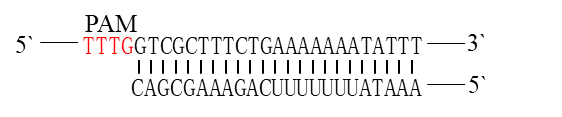 |
| **2** | **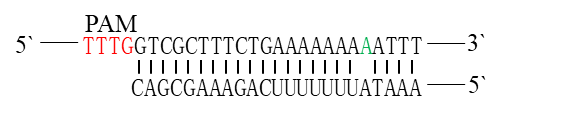** |
| **3** | **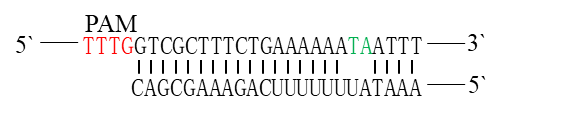** |
| **4** | **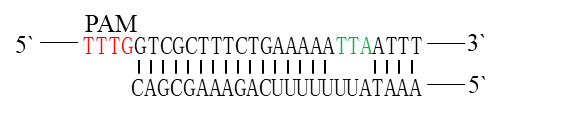** |
| **5** | **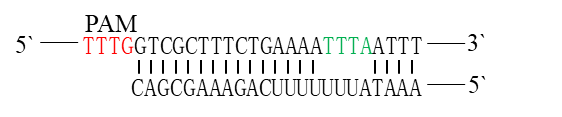** |
| **6** | **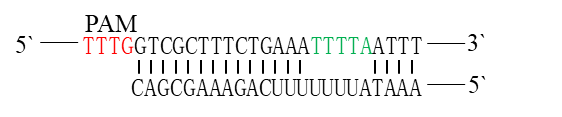** |
| **7** | **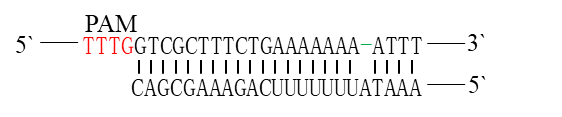** |
| 8 | 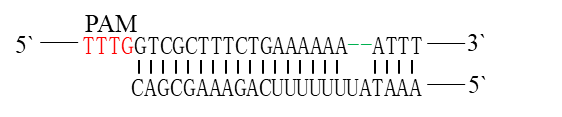 |
| 9 | 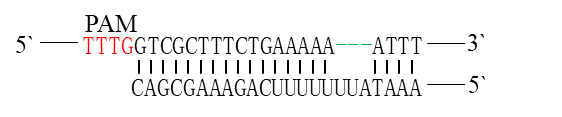 |
| 10 | 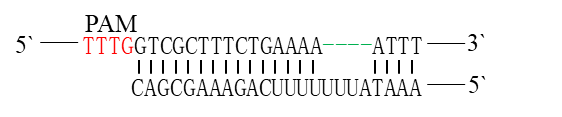 |
| 11 | 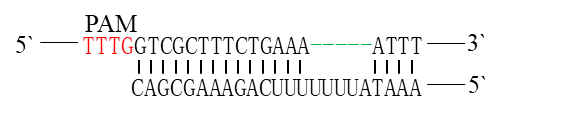 |

PAM sequences are colored in red.

Altered and missing bases in green.

**TABLE S2 | LAMP-CRISPR/Cas12a Assay detection of *Bursaphelenchus xylophilus* from samples +, detected; −, not detected.**

| Code | | Host | Geographical origin | LAMP-CRISPR/Cas12a Assay | PCR | Baermann funnel separation |
| --- | --- | --- | --- | --- | --- | --- |
| 1 | *P. massoniana* | | Guangxi | - | - | - |
| 2 | *P. massoniana* | | Guangxi | + | + | + |
| 3 | *P. massoniana* | | Guangxi | + | + | + |
| 4 | *P. massoniana* | | Guangxi | + | + | + |
| 5 | *P. massoniana* | | Guangxi | - | - | - |
| 6 | *P. massoniana* | | Guangxi | + | + | + |
| 7 | *P. massoniana* | | Guangxi | + | + | + |
| 8 | *P. massoniana* | | Guangxi | + | + | + |
| 9 | *P. massoniana* | | Guangxi | + | + | + |
| 10 | *P. massoniana* | | Guangxi | + | + | + |
| 11 | *P. massoniana* | | Guangxi | - | - | - |
| 12 | *P. massoniana* | | Guangxi | - | - | - |
| 13 | *P. massoniana* | | Guangxi | - | - | - |
| 14 | *P. massoniana* | | Anhui | + | + | + |
| 15 | *P. massoniana* | | Anhui | + | + | + |
| 16 | *P. massoniana* | | Anhui | + | + | + |
| 17 | *P. massoniana* | | Jiangsu | + | + | + |
| 18 | *P. massoniana* | | Jiangsu | + | + | + |
| 19 | *P. massoniana* | | Jiangsu | + | + | + |
| 20 | *P. massoniana* | | Jiangsu | + | + | + |
| 21 | *P. massoniana* | | Jiangsu | + | + | + |
| 22 | *P. massoniana* | | Hubei | + | + | + |
| 23 | *P. massoniana* | | Hubei | + | + | + |
| 24 | *P. massoniana* | | Hubei | + | + | + |
| 25 | *P. massoniana* | | Hubei | + | + | + |
| 26 | *P. koraiensis* | | Liaoning | + | + | + |
| 27 | *P. koraiensis* | | Liaoning | + | + | + |
| 28 | *P. koraiensis* | | Liaoning | + | + | + |
| 29 | *P. koraiensis* | | Liaoning | + | + | + |
| 30 | *P. koraiensis* | | Liaoning | + | + | + |
| 31 | *P. koraiensis* | | Liaoning | - | - | - |
| 32 | *P. koraiensis* | | Liaoning | - | - | - |
| 33 | *P. koraiensis* | | Liaoning | + | + | + |
| 34 | *P. koraiensis* | | Liaoning | + | + | + |
| 35 | *P. koraiensis* | | Liaoning | + | + | + |
| 36 | *P. koraiensis* | | Liaoning | + | + | + |
| 37 | *P. koraiensis* | | Liaoning | + | + | + |
| 38 | *P. koraiensis* | | Liaoning | + | + | + |
| 39 | *P. koraiensis* | | Liaoning | - | - | - |
| 40 | *P. koraiensis* | | Liaoning | + | + | + |
| 41 | *P. koraiensis* | | Liaoning | + | + | + |
| 42 | *P. koraiensis* | | Liaoning | + | + | + |
| 43 | *P. koraiensis* | | Liaoning | + | + | + |
| 44 | *P. koraiensis* | | Liaoning | + | + | + |
| 45 | *P. koraiensis* | | Liaoning | + | + | + |
| 46 | *P. koraiensis* | | Liaoning | + | + | + |
